# Type-specific sensitivity versus detection breadth in HPV diagnostics: improved analytical detection of HPV16/18 by nested PCR

**DOI:** 10.1007/s00705-026-06691-5

**Published:** 2026-07-20

**Authors:** Yago Tomaz Vieira da Silva, Larissa Alves Honorato Ferreira, Samara Fontes de Lima Gomes, Beatriz Helena Dantas Rodrigues De Albuquerque, Maryana Thalyta Ferreira Câmara De Oliveira, Alice Dantas Leite, José Veríssimo Fernandes, Ricardo Ney Oliveira Cobucci, Daniel Carlos Ferreira Lanza

**Affiliations:** 1 Applied Molecular Biology Laboratory (LAPLIC), Department of Biochemistry, Federal University of Rio Grande do Norte, Campus Universitário, Lagoa Nova, Natal, RN CEP 59078-970 Brazil; 2 Januário Cicco Maternity School, Federal University of Rio Grande do Norte, Natal, RN Brazil; 3 Department of Microbiology and Parasitology, Federal University of Rio Grande do Norte, Natal, RN Brazil; 4 Graduate Program in Sciences Applied to Women’s Health, Federal University of Rio Grande do Norte, Natal, RN Brazil; 5 Graduate Program in Biotechnology, Potiguar University, Natal, RN Brazil

## Abstract

Accurate detection of high-risk human papillomavirus (HPV) is central to cervical cancer screening. Current diagnostic strategies increasingly prioritize broad-spectrum assays to maximize genotype coverage. However, theoretical genotype breadth does not necessarily translate into effective sample-level detection when clinically dominant targets are present at low copy number. Here, we evaluated this analytical trade-off using conventional PCR as a controlled experimental framework. Type-specific nested PCR assays targeting HPV16 and HPV18 were developed and applied to 41 cervical samples. Nested PCR showed markedly higher relative analytical sensitivity than single-round amplification and produced a greater proportion of specific and interpretable detections than the broad-spectrum consensus systems tested here (MY09/11 and GP5/6). HPV16 and/or HPV18 DNA was detected by nested PCR in 22/41 samples, whereas MY09/11 and GP5/6 detected 11/41 and 2/41 samples, respectively, under the tested conditions. These findings indicate that broad genotype coverage and effective detection of positive samples are distinct analytical properties. In settings where prevalent HPV targets occur at low abundance, a highly sensitive genotype-focused assay may reveal more target-positive samples than a broader but less sensitive consensus approach. The results are not intended to replace clinically validated broad-spectrum screening assays, but they support explicit preservation of per-target analytical sensitivity in reflex testing and future multi-target HPV assay design.

**Supplementary Information:**

The online version contains supplementary material available at 10.1007/s00705-026-06691-5.

## Introduction

Human papillomavirus (HPV) infection is the central etiological factor in cervical cancer, making accurate molecular detection of HPV DNA essential for screening, clinical management, and epidemiological surveillance [1, 2]. Increasing demand for scalable and cost-effective testing has driven a progressive shift toward molecular assays capable of detecting multiple HPV genotypes simultaneously [2, 3]. Broad-spectrum PCR systems and, more recently, multiplex platforms enable simultaneous detection of diverse viral targets in a single reaction, improving operational efficiency and throughput in large-scale screening settings [3, 4].

This transition is often interpreted as an increase in diagnostic coverage because a larger number of genotypes can be targeted simultaneously [5, 6]. However, theoretical genotype breadth and effective sample-level detection are not equivalent properties. Amplification of several targets under shared reaction conditions may produce heterogeneous amplification efficiency across genotypes, particularly when viral load is low or sequence variability is present [7, 8]. Therefore, a broader assay may fail to reveal target-positive samples if sensitivity for the most prevalent or clinically relevant targets is compromised.

This issue is particularly relevant for HPV16 and HPV18, the most clinically significant high-risk genotypes worldwide, although the distribution of high-risk HPV types varies across populations [9, 10]. For patient-level detection, the relevant practical question is not only how many genotypes an assay is designed to detect, but whether it retains sufficient per-target sensitivity to detect the dominant genotypes when viral DNA is present near the detection limit [11, 12]. Thus, a genotype-focused strategy may, in some analytical contexts, identify more HPV-positive samples for selected prevalent genotypes than a broad-spectrum assay with lower per-target sensitivity.

Nested PCR is a well-established strategy for enhancing analytical sensitivity and specificity when target DNA is present near the detection limit [13, 14]. By employing sequential amplification with internal primers, type-specific nested assays maximize signal recovery under controlled conditions and provide a suitable framework for evaluating the relationship between detection breadth and effective target detection [13–15]. Conventional endpoint PCR further offers a controlled analytical environment, allowing direct visualization of amplification products with minimal influence from fluorescence background or probe-dependent effects and enabling stringent implementation of nested workflows [16].

In this study, we used conventional PCR as a controlled experimental model to compare a highly sensitive type-specific nested PCR approach for HPV16 and HPV18 with broad-spectrum consensus PCR protocols [17, 18]. We did not aim to replace clinically validated commercial HPV assays or to estimate clinical screening performance. Rather, we tested the narrower analytical hypothesis that greater genotype breadth may not maximize effective detection of HPV16/18-positive samples when viral DNA is present at low abundance. This distinction is relevant to reflex analytical workflows and to the development of future multi-target molecular diagnostic systems in which breadth must not be gained at the expense of per-target sensitivity.

## Materials and methods

### Primer design for type-specific nested PCR

Complete genome sequences of Human Papillomavirus (HPV) types 16 and 18 were retrieved from the NCBI Nucleotide database in October 2022. All available complete genomes were included (accession numbers in Supplementary Materials 1–2). Genome orientation was standardized using MARS [19], followed by multiple sequence alignment with MAFFT [20].

Primers were designed in Geneious Prime v9.0.5 targeting conserved regions of the HPV16 E6/E7 genes and the HPV18 E1 gene. Specificity was evaluated using Primer-BLAST [21] against human, viral, bacterial, fungal, and apicomplexan genomes. Primer–dimer formation and secondary structures were assessed using AutoDimer [22].

### General PCR plastics, equipment, electrophoresis, and contamination-control procedures

All PCR assays were performed in sterile 0.2-mL PCR microtubes using aerosol-resistant filtered tips appropriate for each pipetting volume. PCR tubes and filtered tips were from Kasvi. The 0.2-mL PCR tubes corresponded to standard flat-cap PCR microtubes, and filtered tips included universal aerosol-resistant tips in the volume ranges required for reaction setup, including 1–200 µL tips. Amplifications were performed in a LifeTouch TC-96/G/H(b)B thermal cycler (Bioer).

PCR products were analyzed by agarose gel electrophoresis. Unless otherwise specified, products were resolved on 1.5% (w/v) agarose gels prepared in 1× TAE buffer and stained with ethidium bromide. Band visualization was performed under UV light using a UV6 transilluminator (Delpho). Fragment sizes were estimated using the 50-bp or 100-bp DNA ladder (Invitrogen).

To reduce the risk of carryover contamination [23, 24], pre- and post-amplification procedures were conducted in physically separated areas using dedicated equipment and aerosol-resistant filtered tips. Run-specific negative controls were included in all amplification experiments, and reportable bands were confirmed in independent experiments.

### Type-specific nested PCR

Assay validation was performed using genomic DNA from SiHa (HPV16-positive) and HeLa (HPV18-positive) cell lines [25, 26]. All type-specific nested PCR assays were performed in singleplex, with HPV16 and HPV18 amplified in independent reactions.

PCR reactions (20 µL) contained 1× reaction buffer, 1.5 mM MgCl₂, 0.2 mM dNTPs, 1 µM of each primer, 1 U Taq DNA polymerase (Ludwig Biotec), and 1 µL template DNA. Cycling conditions were: 94 °C for 2 min; 30 cycles of 94 °C for 1 min, 52 °C for 1 min, and 72 °C for 1 min; final extension at 72 °C for 5 min.

### Consensus PCR assays

Broad-spectrum HPV detection was performed using MY09/MY11 consensus primers and the original GP5/6 primer system. The MY09/MY11 assay was based on the protocol described by Manos et al. [17], whereas the GP5/6 assay was based on the general primer system described by Snijders et al. [18].

Before application to clinical samples, both consensus assays were optimized within the same laboratory workflow used for the type-specific assays, including reagent source, thermocycler, template input, electrophoresis system, and positivity criteria, but using assay-specific cycling parameters as detailed below. Temperature-gradient PCR assays were performed using HPV-positive control templates from SiHa and HeLa genomic DNA to identify the annealing condition that provided the best balance between expected-size amplification, analytical sensitivity, and specificity, while minimizing nonspecific products. Under the reagent, thermocycler, template, and electrophoresis conditions used in this study, 53 °C provided the most reproducible amplification profile for the consensus assays and was therefore used for all clinical-sample comparisons.

PCR reactions were prepared in a final volume of 20 µL and contained 1× reaction buffer, 1.5 mM MgCl₂, 0.2 mM dNTPs, 1 µM of each primer, 1 U Taq DNA polymerase (Ludwig Biotec), and 1 µL template DNA. Cycling conditions were 94 °C for 5 min, followed by 40 cycles of 94 °C for 1 min, 53 °C for 2 min, and 72 °C for 1.5 min, with a final extension at 72 °C for 5 min.

### Analytical sensitivity assessment

Relative analytical sensitivity was evaluated using tenfold serial dilutions of genomic DNA extracted from SiHa and HeLa cells. This cell-derived DNA model was used to compare assay performance under the same template background, while preserving extraction-associated losses and genomic DNA context. Diluted templates were subjected to first-round PCR, and the most diluted detectable products were used as templates for the nested reaction.

The relative analytical dilution endpoint was defined as the highest dilution yielding a discrete amplicon of the expected size after gel electrophoresis. Because the dilution series was not prepared from independently quantified HPV copy-number standards, the results were interpreted as relative analytical sensitivity, not as an absolute copy-number limit of detection [27].

### Clinical samples

The study was approved by the institutional ethics committee (CAAE 30679220.9.0000.5292) and conducted in accordance with national regulations for research involving human subjects.

Endocervical samples were obtained by cytobrush collection during routine gynecological care at Maternidade Escola Januário Cicco (Natal, Brazil), a hospital-based follow-up setting. The study used a convenience set of samples from women with and without cervical lesions, without prior selection according to cytological findings. Samples were stored at −80 °C until processing.

Because the objective was to compare analytical detection performance among PCR strategies, the cohort was analyzed as an analytical comparison set rather than as a clinically stratified screening cohort. Clinical variables required for prevalence estimation, CIN2+ stratification, predictive-value analysis, or diagnostic accuracy assessment against a validated clinical HPV reference assay were therefore not prospectively collected in a uniform manner for all participants.

### DNA extraction and quality assessment

Genomic DNA was extracted using the Wizard Genomic DNA Purification Kit (Promega) according to the manufacturer's protocol. DNA concentration and purity were assessed by spectrophotometry (NanoVue Plus, GE Healthcare). DNA integrity and absence of gross PCR inhibition were confirmed by amplification of the human actin gene (838 bp) using previously described primers [28]. This assay was used as a sample-quality control and not as a proxy for the detectability of low-copy HPV targets.

### Definition of amplification positivity

PCR products were analyzed by agarose gel electrophoresis. Each assay was performed in two independent amplification experiments, and gel images were independently evaluated by two investigators blinded to sample identity. Reportable positive calls showed complete concordance between independent experiments and between investigators. A sample was considered positive only when a discrete band at the expected size was observed in both independent experiments and concordantly classified as positive by both investigators [13, 29]. Smears, faint bands, non-reproducible products, discordant interpretations, or bands of unexpected size were classified as nonspecific and were not considered reportable positive detections.

### Statistical analysis

Detection rates were calculated as proportions with 95% confidence intervals (Wilson method) [30]. Differences in detection yield between methods applied to the same samples were evaluated using McNemar’s exact test for paired data [31]. Matched odds ratios were calculated to quantify discordant outcomes. Statistical analyses were performed using R software (version 4.3.1) [32], and two-sided p-values < 0.05 were considered statistically significant.

## Results

### Primer design and *in silico* validation

Multiple sequence alignment of 634 complete HPV16 genomes and 146 complete HPV18 genomes identified conserved regions suitable for type-specific primer design. Conserved sequences within the E6/E7 genes were selected as targets for HPV16 detection and genotyping, whereas the E1 gene was selected for HPV18 because it was conserved across the sequences analyzed. The main characteristics of the primer sets are summarized in Table 1.

**Table 1:** Main characteristics of the primers used in this study

Primer name	Primer sequence (5' - 3')	Amplicon (bp)	Tm (°C)	Target	Thermal cycling
Actin_F	TGGAAAAGATCTGGCACC	838	54.44	Actin	1 Cycle: 94 °C for 2 minutes, 30 Cycles: 94 °C for 40 seconds, 53 °C for 40 seconds, 72 °C for 40 seconds, 1 Cycle: 72 °C for 3 minutes.
Actin_R	TCCTGTTTGCTGATCCAC		54.48		
MY09	CGTCCAAAAGGAAACTGAGC	~450	57.31	L1	1 Cycle: 94 °C for 5 minutes, 40 Cycles: 94 °C for 1 minute, 53 °C for 2 minutes, 72 °C for 1.5 minutes
MY11	GCACAGGGACATAACAATGG		56.77		
GP5	TTTGTTACTGTGGTAGATAC	~150	49.80		
GP6	GAAAAATAAACTGTAAATCA		44.88		
YTMZ 16-01_F*	GTGTACTGCAAGCAACAGT	594	56.08	E6/E7	1 Cycle: 94 °C for 2 minutes, 30 Cycles: 94 °C for 1 minute, 52 °C for 1 minute, 72 °C for 1 minute, 1 Cycle: 72 °C for 5 minutes.
YTMZ 16-01_R*	TGTCTACGTGTGTGCTTTG		55.82		
YTMZ 18-01_F*	AACCCAACAATAGCAGAAGG	716	55.62	E1	
YTMZ 18-01_R*	CCTGTATTTGCTGGTCCAC		55.89		
YTMZ 16-02_F*	ATTAGGTGTATTAACTGTC	282	46.35	E6/E7	
YTMZ 16-02_R*	CTGGACCATCTATTTCAT		47.99		
YTMZ 18-02_F*	ACCACCAAAATTGCGAAGTAG	242	57.08	E1	
YTMZ 18-02_R*	CATTGCTGTTGCTGTCTGC		57.9		

^*^ Primers developed in this study

The expected amplicon sizes for the first PCR round were 594 bp for HPV16 and 716 bp for HPV18, while the nested PCR step generated internal fragments of 282 bp (HPV16) and 242 bp (HPV18). *In silico* specificity analysis predicted annealing of the primer sets to their respective HPV genotypes, with no predicted cross-reactivity with human, bacterial, fungal, or other viral sequences.

### Standardization of the nested PCR protocol

**Fig. 1:**
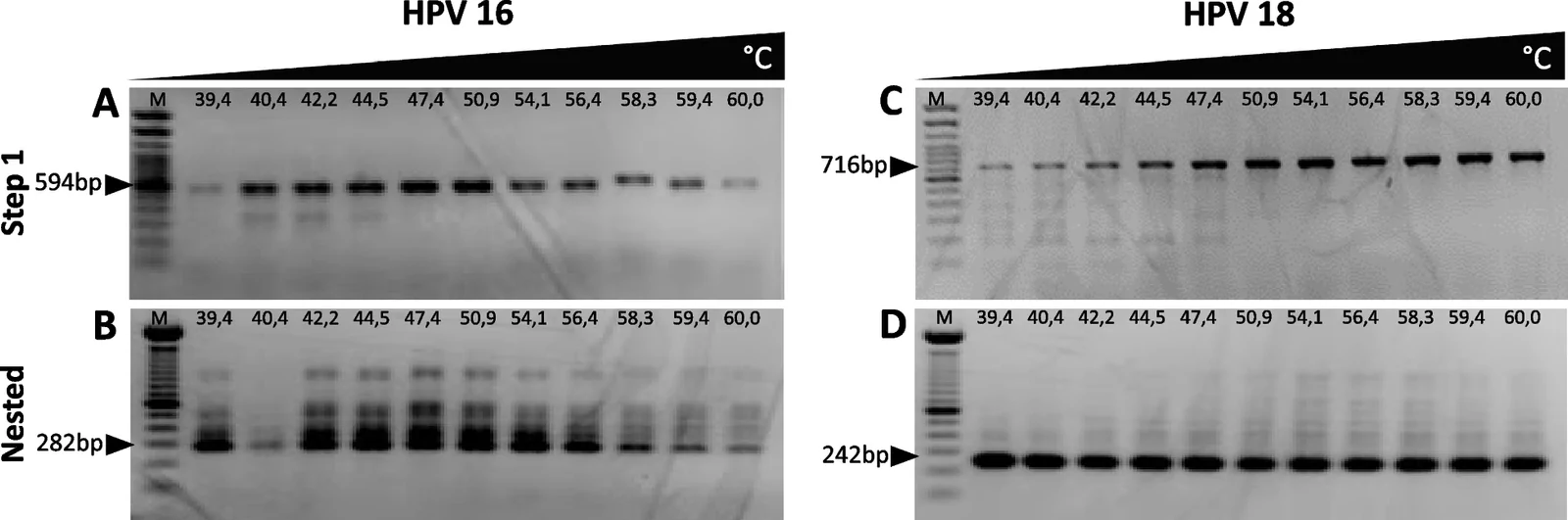
Optimization of annealing temperature for type-specific nested PCR assays. Agarose gel electrophoresis showing amplification products obtained across an annealing temperature gradient for primers developed in this study. (**A**) First-round PCR amplification of HPV16 from SiHa genomic DNA using primers YTMZ 16-01_F/R. (**B**) Nested PCR amplification of HPV16 using primers YTMZ 16-02_F/R. (**C**) First-round PCR amplification of HPV18 from HeLa genomic DNA using primers YTMZ 18-01_F/R. (**D**) Nested PCR amplification of HPV18 using primers YTMZ 18-02_F/R. Annealing temperatures increase across lanes as indicated. Arrows denote expected amplicon sizes. M, 100-bp DNA ladder

The type-specific nested PCR assays were validated using genomic DNA from SiHa (HPV16-positive) and HeLa (HPV18-positive) cell lines. Both targets produced amplification products of the expected sizes in the first and second amplification rounds (Fig. 1).

Optimization experiments identified an annealing temperature of 52 °C as providing the best balance between analytical sensitivity and specificity across all primer sets. Under these conditions, amplification was robust, reproducible, and consistently yielded discrete bands without nonspecific products, confirming reliable performance of the nested PCR workflow (Table 1).

### Analytical sensitivity of nested PCR compared with consensus PCR methods

**Fig. 2:**
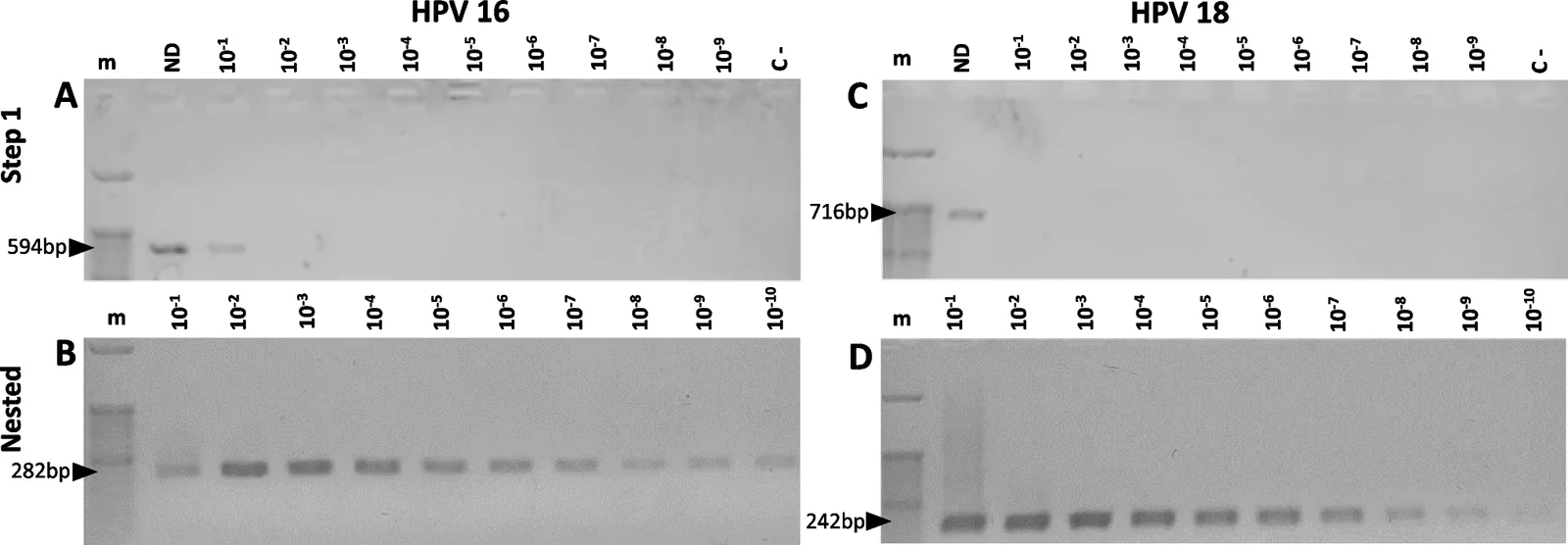
Analytical sensitivity of type-specific nested PCR assays. Assessment of analytical sensitivity using tenfold serial dilutions of genomic DNA from HPV-positive cell lines. (**A**) First-round PCR detection of HPV16 from SiHa DNA using primers YTMZ 16-01_F/R. (**B**) Nested PCR detection of HPV16 using primers YTMZ 16-02_F/R. (**C**) First-round PCR detection of HPV18 from HeLa DNA using primers YTMZ 18-01_F/R. (**D**) Nested PCR detection of HPV18 using primers YTMZ 18-02_F/R. Dilution factors are indicated above each lane. Arrows indicate expected amplicon sizes, and templates used in nested reactions are highlighted. ND, non-diluted DNA. C-, negative control. m, 50-bp DNA ladder

Analytical sensitivity was evaluated using tenfold serial dilutions of genomic DNA extracted from SiHa and HeLa cells (Fig. 2). In the first-round PCR, HPV16 was detectable only up to a 10⁻^1^ dilution of SiHa DNA, whereas HPV18 amplification from HeLa DNA was observed exclusively in the undiluted template, indicating limited detection sensitivity at this stage.

In contrast, the nested PCR step markedly increased relative analytical sensitivity. HPV16 and HPV18 were detectable in serial dilutions down to 10⁻^1^⁰ and 10⁻⁹, respectively, representing a substantial improvement in detection capability relative to single-round amplification. As expected, band intensity decreased with increasing dilution, consistent with lower template abundance. Because serial dilutions were performed on extracted genomic DNA, these values represent relative analytical dilution endpoints under the tested conditions and were not converted into absolute HPV genome copy numbers.

For comparison, consensus PCR protocols using MY09/11 and GP5/6 primers were evaluated within the same laboratory workflow and interpretation criteria as the nested assays, while using their assay-specific primer sets and cycling parameters. Both methods produced amplification from positive control templates. However, nonspecific amplification products were frequently observed, and detection in serially diluted templates was lower than that achieved with the type-specific nested PCR assays. Under the tested conditions, no consensus PCR condition combined absence of nonspecific amplification with detection sensitivity comparable to the nested PCR assays.

### Detection of HPV16 and HPV18 in clinical samples

**Fig. 3:**
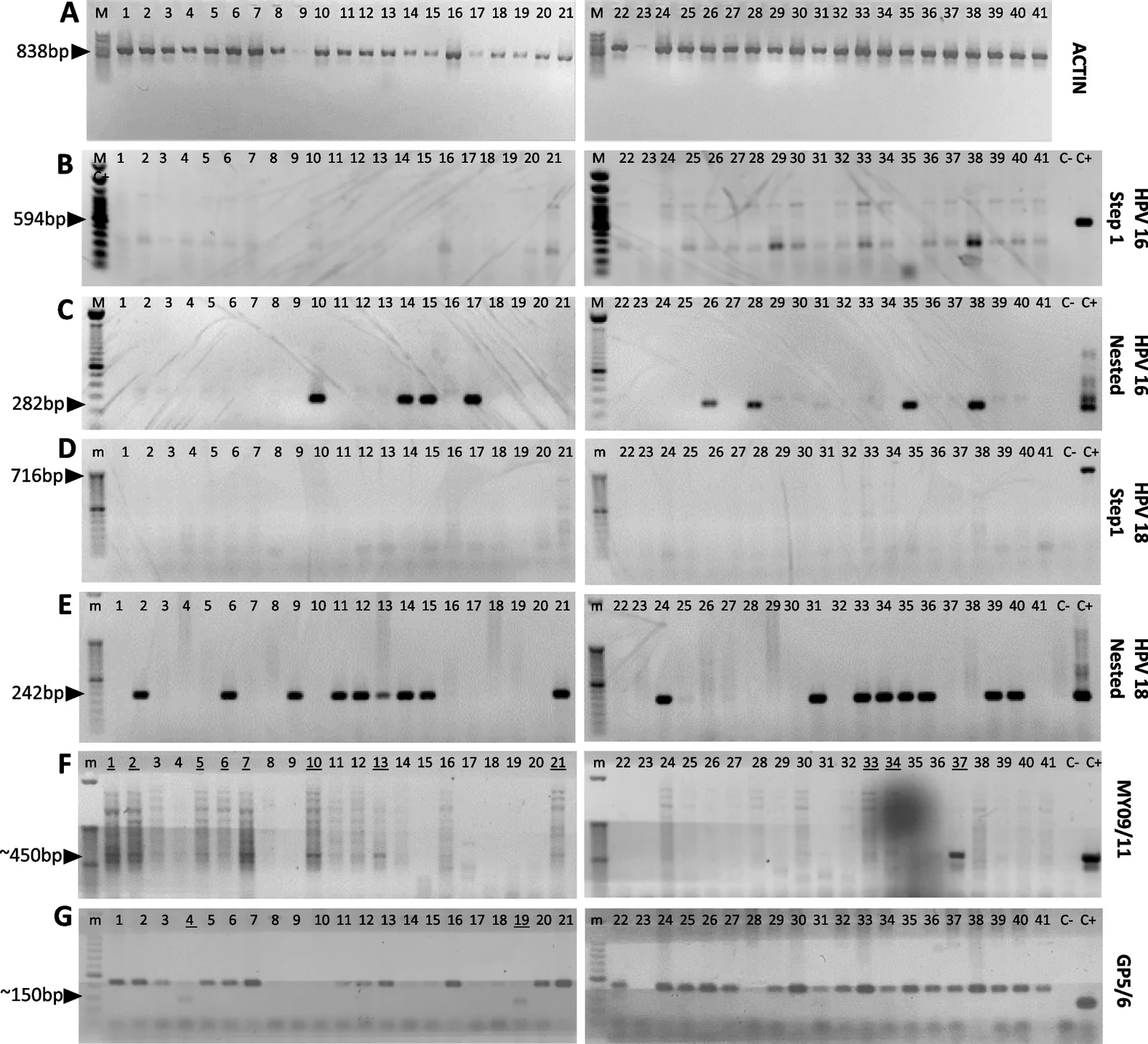
Comparative detection of HPV16 and HPV18 in clinical endocervical samples. Representative agarose gel electrophoresis images showing: (**A**) Amplification of the human actin gene as a DNA-integrity and PCR-inhibition control; (**B**) First-round HPV16 PCR using primers YTMZ 16-01_F/R; (**C**) Nested HPV16 PCR using primers YTMZ 16-02_F/R; (**D**) First-round HPV18 PCR using primers YTMZ 18-01_F/R; (**E**) Nested HPV18 PCR using primers YTMZ 18-02_F/R; (**F**) Broad-spectrum HPV detection using MY09/MY11 consensus primers; and (**G**) Broad-spectrum HPV detection using GP5/6 consensus primers. Arrows indicate the expected amplicon sizes. M, 100-bp DNA ladder; m, 50-bp DNA ladder. C-, negative control; C+, positive control. In panels F and G, only underlined lane numbers correspond to samples classified as reportable positives according to the predefined criteria

A total of 41 endocervical samples from women aged 19–46 years (mean 35.2 ± 7.8 years) were analyzed (Supplementary Material 3 - Graph 1). Successful amplification of the human actin gene was observed in all samples (Fig. 3A), confirming the presence of amplifiable human genomic DNA and the absence of gross PCR inhibition under the extraction and amplification conditions used. Thus, all specimens were considered suitable for comparative HPV PCR analysis. Available routine cytology is presented sample by sample in Supplementary Material 3. Ten specimens were reported as unsatisfactory for cytology. Among the 31 satisfactory cytology reports, findings were mainly inflammatory and/or microbiological, including Lactobacillus, Candida, cocci, other bacilli, or supracytoplasmic bacilli suggestive of Gardnerella/Mobiluncus, with one report of immature squamous metaplasia and no cytological diagnosis of intraepithelial lesion or malignancy. Histopathological CIN grade, cervical cancer diagnosis, and a uniformly applied clinical HPV reference assay were not available for all specimens; therefore, positivity according to CIN/cancer status or reference-test accuracy could not be analyzed.

No clinical sample showed detectable amplification for HPV16 or HPV18 in the first-round type-specific PCR (Fig. 3B and D). After nested PCR, HPV16 and/or HPV18 DNA was detected in 22/41 samples (53.7%; 95% CI: 38.7–67.9). HPV18 was detected in 17/41 samples (41.5%; 95% CI: 27.8–56.6), HPV16 in 8/41 samples (19.5%; 95% CI: 10.2–34.0), and HPV16/HPV18 coinfection was observed in three samples. Detection yields are summarized in Table 2.

**Table 2:** Detection yield of type-specific nested PCR and consensus PCR methods in 41 clinical cervical samples

Method	Target	Positive samples (n/41)	Detection rate (%)*	95% confidence interval
Type-specific nested PCR	HPV16	8/41	19.5	10.2–34.0
Type-specific nested PCR	HPV18	17/41	41.5	27.8–56.6
Type-specific nested PCR (overall)	HPV16 and/or HPV18	22/41	53.7	38.7–67.9
Consensus PCR (MY09/11)	Broad HPV detection	11/41	26.8	15.7–41.9
Consensus PCR (GP5/6)	Broad HPV detection	2/41	4.9	1.3–16.1

^*^Detection rates represent the proportion of samples showing a discrete band at the expected size. Smears, faint signals, or bands of unexpected size were classified as nonspecific and excluded from positive detection counts. Consensus PCR assays detect multiple HPV genotypes, whereas type-specific nested PCR assays target HPV16 and HPV18

Consensus PCR using MY09/MY11 primers generated expected-size bands in 11/41 samples (26.8%; 95% CI: 15.7–41.9) (Fig. 3F). GP5/6 PCR generated expected-size bands in 2/41 samples (4.9%; 95% CI: 1.3–16.1) (Fig. 3G). Nonspecific amplification products were observed in both consensus PCR assays.

Because all assays were applied to the same clinical samples, paired comparisons were performed to evaluate differences in reportable detection yield. For the nested PCR versus MY09/MY11 comparison, nested PCR was positive while MY09/MY11 was negative in 15 samples, whereas MY09/MY11 was positive while nested PCR was negative in four samples (matched odds ratio = 3.75; McNemar exact p = 0.019). For the nested PCR versus GP5/6 comparison, nested PCR was positive while GP5/6 was negative in 22 samples, whereas GP5/6 was positive while nested PCR was negative in two samples (matched odds ratio = 11.0; McNemar exact p = 3.6 × 10⁻^5^) (Table 3).

**Table 3:** Paired comparison of reportable detection yield between type-specific nested HPV16/18 PCR and broad-spectrum consensus PCR assays under the tested analytical conditions

Comparison	Nested+/consensus−	Nested−/consensus+	Matched odds ratio	McNemar *p*-value*
Nested vs MY09/11	15	4	3.75	0.019
Nested vs GP5/6	22	2	11.0	3.6×10⁻^5^

McNemar exact test was used for paired comparison of detection outcomes

## Discussion

HPV16 and HPV18 account for a substantial proportion of cervical cancer cases worldwide, making reliable detection of these high-risk genotypes central to molecular diagnostics, screening, and epidemiological surveillance [2, 3, 8–10]. In this study, we developed type-specific nested PCR assays for HPV16 and HPV18 and compared their analytical detection with first-round type-specific PCR and with the broad-spectrum consensus systems tested here. The study addressed a specific methodological question: whether broader genotype coverage necessarily results in greater effective sample-level detection of clinically dominant HPV genotypes.

Our findings indicate that this assumption does not always hold. Broad-spectrum assays such as MY09/MY11 and GP5/6 were designed to increase genotype coverage [17, 18]. However, effective detection also depends on the analytical sensitivity achieved for each target, particularly when viral DNA is present at low abundance or when amplification efficiency differs among genotypes [5, 7, 12]. In the clinical samples analyzed here, first-round type-specific PCR did not detect HPV16 or HPV18, whereas the consensus PCR assays detected expected-size bands in a subset of samples. This shows that type-specific primer design alone was not responsible for the increased detection.

The main gain was observed when type-specific targeting was combined with nested amplification. After the nested step, HPV16 and/or HPV18 DNA was detected in more samples than by first-round type-specific PCR or by the single-round consensus assays. Therefore, the advantage observed here should be interpreted as the effect of a high-sensitivity, genotype-focused nested workflow, not simply as an effect of type-specific primers. This distinction is central: broad-spectrum PCR provides wider theoretical genotype coverage, but a nested type-specific strategy may provide greater effective detection for selected high-prevalence targets when their abundance is below the detection threshold of single-round assays. In the present cohort, HPV16/HPV18 co-infection was observed in 3/41 samples (7.3%) and in 3/22 nested-positive samples (13.6%). This finding should be interpreted cautiously because the cohort was small and not clinically stratified. Reported frequencies of multiple HPV infections, including combinations involving HPV16 and/or HPV18, vary substantially across populations, age groups, testing platforms, and lesion-enriched versus screening settings; single-type infections are generally predominant in broad screening cohorts, whereas HPV16/18-positive cohorts referred to colposcopy may show higher proportions of multiple-type co-infection [33, 39].

Nested PCR enhances detection through sequential amplification with internal primers, increasing target recovery when template availability is low [13]. Consistent with this mechanism, samples that were negative by first-round amplification or consensus PCR produced expected-size products after the nested step. This pattern is also consistent with previous comparisons showing that consensus PCR and type-specific PCR strategies may differ in their ability to detect oncogenic HPV infections [7]. In the present study, this difference was most evident for HPV16/18 detection under low-template conditions, supporting the distinction between genotype breadth and per-target sensitivity.

The use of conventional endpoint PCR was deliberate. It provided a controlled analytical framework in which amplification products could be directly visualized by gel electrophoresis, using the same reagent, thermocycler, template, and interpretation criteria across assays [16]. This design was not intended to evaluate or replace optimized commercial HPV screening platforms, which require clinical validation according to established performance criteria [5, 8]. Rather, it isolated an analytical issue relevant to assay design: expanding genotype coverage should not be assumed to preserve equivalent sensitivity for each individual target.

Contamination control was addressed through physical separation of pre- and post-amplification areas, dedicated materials, aerosol-resistant filter tips, run-specific negative controls, and confirmation of reportable bands in independent experiments [23, 34]. No evidence of contamination was observed in negative controls. Although a formal carryover challenge experiment would strengthen future validation, this does not compromise the comparative analytical conclusion of the present study, because reported detections were based on reproducible expected-size bands under predefined criteria.

Some limitations define the scope of interpretation. *In silico* specificity analysis against viral and non-viral databases does not replace empirical cross-reactivity testing against a broad HPV genotype panel [21]. The HPV18 assay targets E1, a region that may be affected by integration-associated disruption in some infections [35]. However, HPV integration does not necessarily imply complete loss of all E1-containing viral molecules in a clinical specimen, because episomal, integrated, and mixed viral forms may coexist. Therefore, E1 disruption represents a potential source of false negativity mainly in specimens containing exclusively disrupted HPV18 genomes affecting the primer-binding region. Within the present analytical framework, E1 remained a conserved and highly detectable target, although future clinical validation may benefit from inclusion of an additional HPV18 E6/E7 target.

Additional limitations relate to sensitivity calibration and clinical extrapolation. The dilution experiments provide relative analytical sensitivity under cell-derived genomic DNA conditions, not an absolute copy-number limit of detection [27]. In addition, because this was an analytical comparison study, clinical variables required for prevalence estimation, CIN2+ stratification, predictive-value analysis, or diagnostic accuracy assessment against a validated clinical HPV reference assay were not prospectively collected in a uniform manner for all participants. These limitations restrict clinical extrapolation, but they do not invalidate the primary analytical finding that, under the tested conditions, the type-specific nested workflow detected more reportable HPV16/18-positive samples than the broad-spectrum consensus assays evaluated. Therefore, the present study should not be interpreted as a VALGENT-type clinical validation study [36]. Instead, it represents an analytical comparison of reportable detection yield under controlled conventional PCR conditions.

These findings are relevant to large-scale and comprehensive HPV studies, which require broad, standardized, and clinically validated assays to capture genotype diversity, as illustrated by Bekmukhambetov et al. [37]. This is particularly important because HPV genotype distribution varies across populations, as shown by Jeudin et al. [38]. Our results do not argue against comprehensive HPV testing. Instead, they indicate that broad assays and future multiplex platforms should be designed so that expansion of genotype coverage does not reduce per-target sensitivity for dominant genotypes. High-sensitivity genotype-focused components may therefore be useful in assay-development pipelines or as reflex analytical tools for samples that are negative or inconclusive by broad-spectrum methods but remain clinically or epidemiologically relevant.

## Conclusion

Type-specific nested PCR provided higher analytical detection of HPV16 and HPV18 than the broad-spectrum consensus PCR protocols tested under the same experimental conditions. These findings show that theoretical genotype breadth does not necessarily ensure effective sample-level detection when clinically dominant targets are present at low abundance. Genotype-focused high-sensitivity strategies may therefore complement, rather than replace, comprehensive HPV testing by improving detection of selected prevalent targets in analytically challenging samples. Further validation using quantified standards, broader cross-reactivity panels, carryover studies, and clinically characterized cohorts with CIN2+ endpoints is required before clinical screening utility can be inferred.

## Supplementary Information

Below is the link to the electronic supplementary material.

Supplementary file1 (TXT 49 KB)

Supplementary file2 (TXT 11 KB)

Supplementary file3 (DOCX 44 KB)

## Data Availability

No datasets were generated or analysed during the current study.
