# Supplementary material for: Type-specific sensitivity versus detection breadth in HPV diagnostics: improved analytical detection of HPV16/18 by nested PCR
Source: Arch Virol. 2026 Jul 20;171(8):230. doi: 10.1007/s00705-026-06691-5 (PMC13385011; doi:10.1007/s00705-026-06691-5)
Supplement: Supplementary file 3 — Supplementary file3 (DOCX 44 KB) [file 705_2026_6691_MOESM3_ESM.docx]

**Supplementary Material 3. Available demographic, routine cytological, and nested HPV16/18 analytical information for the 41 endocervical samples.**

Note. Available clinical correlates consisted of age and routine cervical cytology. Histopathological CIN grade, cervical cancer diagnosis, and a validated HPV reference assay were not uniformly available for all specimens. Therefore, this analytical sample set does not support CIN-specific sensitivity/specificity estimates or predictive-value analysis. Sample-level nested-PCR calls are provided only as analytical HPV16/18 results and should not be interpreted as clinical disease-status classifications.

**Summary of available clinical and analytical information:**

Number of endocervical samples: 41.

Age: mean 35.2 ± 7.8 years; range 19-46 years.

Routine cytology: reported sample by sample below; 10/41 specimens were reported as unsatisfactory for cytology.

Satisfactory cytology reports: among the 31 satisfactory reports, findings were mainly inflammatory and/or microbiological; no cytological diagnosis of intraepithelial lesion or malignancy was recorded.

Nested PCR aggregate results: HPV16, 8/41; HPV18, 17/41; HPV16 and/or HPV18, 22/41; HPV16/HPV18 co-infection, 3/41. Sample-level nested-PCR results are reported in the table below using + for reportable positive and − for negative.

| **No.** | **Internal code** | **Age** | **Routine cytology** | **Nested HPV16** | **Nested HPV18** | **Nested HPV16 and/or 18** |
| --- | --- | --- | --- | --- | --- | --- |
| 1 | LAP | 44 | Without representation of the transformation zone/Inflammatory/ Lactobacillus/ Negative for malignancy | − | − | − |
| 2 | MASF | 37 | Inflammatory/ Lactobacillus/ Negative for malignancy | − | + | + |
| 3 | JLAR | 42 | Inflammatory/ Lactobacillus/ Negative for malignancy | − | − | − |
| 4 | ACJS | 26 | Inflammatory/ Cocci, Other bacilli/ Negative for malignancy | − | − | − |
| 5 | MSSS | 36 | Inflammatory/Lactobacillus/Candida/Negative for malignancy | − | − | − |
| 6 | LCM | 31 | Inflammatory/ Supracytoplasmic bacilli (suggestive of Gardnerella/Mobiluncus)/Negative for malignancy | − | + | + |
| 7 | LSG | 45 | Lacking representation from the transformation zone./Inflammatory/ Lactobacillus/ Negative for malignancy | − | − | − |
| 8 | LPNS | 43 | Inflammatory/ Lactobacillus/Negative for malignancy | − | − | − |
| 9 | RCRP | 45 | Inflammatory/ Lactobacillus/Negative for malignancy | − | + | + |
| 10 | ACAS | 22 | Unsatisfactory sample for cytology | + | − | + |
| 11 | MJS | 42 | Inflammatory/ Lactobacillus/ Negative for malignancy | − | + | + |
| 12 | LDSSO | 26 | Inflammatory/ Immature squamous metaplasia/ Other bacilli/Negative for malignancy | − | + | + |
| 13 | ACS | 28 | Inflammatory/ Lactobacillus/ Negative for malignancy | − | + | + |
| 14 | MTBC | 37 | Inflammatory/ Lactobacillus/Negative for malignancy | + | + | + |
| 15 | PMFN | 37 | Unsatisfactory sample for cytology | + | + | + |
| 16 | ECSO | 43 | Unsatisfactory sample for cytology | − | − | − |
| 17 | KOM | 33 | Unsatisfactory sample for cytology | + | − | + |
| 18 | MBS | 38 | Inflammatory/Other bacilli/Negative for malignancy | − | − | − |
| 19 | ASO | 45 | Unsatisfactory sample for cytology | − | − | − |
| 20 | MCLS | 46 | Unsatisfactory sample for cytology | − | − | − |
| 21 | BMLS | 24 | Inflammatory/ Lactobacillus/ Negative for malignancy | − | + | + |
| 22 | LSNS | 46 | Inflammatory/ Lactobacillus/ Negative for malignancy | − | − | − |
| 23 | MCAC | 40 | Inflammatory/ Lactobacillus/ Negative for malignancy | − | − | − |
| 24 | MRSN | 41 | Inflammatory/ Lactobacillus/ Negative for malignancy | − | + | + |
| 25 | WCC | 33 | Inflammatory/ Lactobacillus/ Negative for malignancy | − | − | − |
| 26 | FEFNS | 36 | Inflammatory/ Lactobacillus/ Negative for malignancy | + | − | + |
| 27 | RKMM | 27 | Inflammatory/Lactobacillus/Candida/Negative for malignancy | − | − | − |
| 28 | ESPS | 27 | Inflammatory/ Lactobacillus/ Negative for malignancy | + | − | + |
| 29 | MJSC | 33 | Inflammatory/ Lactobacillus/ Negative for malignancy | − | − | − |
| 30 | GFT | 31 | Inflammatory/ Lactobacillus/ Negative for malignancy | − | − | − |
| 31 | FLSL | 44 | Inflammatory/Lactobacillus/Candida/Negative for malignancy | − | + | + |
| 32 | MGSM | 19 | Unsatisfactory sample for cytology | − | − | − |
| 33 | DO | 45 | Unsatisfactory sample for cytology | − | + | + |
| 34 | JLB | 27 | Inflammatory/ Lactobacillus/ Negative for malignancy | − | + | + |
| 35 | AKGQ | 41 | Inflammatory/ Supracytoplasmic bacilli (suggestive of Gardnerella/Mobiluncus) | + | + | + |
| 36 | LBP | 31 | Inflammatory/ Cocci, Other bacilli/Negative for malignancy | − | + | + |
| 37 | CLM | 32 | Inflammatory/Other bacilli/Negative for malignancy | − | − | − |
| 38 | GAB | 21 | Unsatisfactory sample for cytology | + | − | + |
| 39 | JFSA | 28 | Atrophy with inflammation/ Lactobacillus/Negative for malignancy | − | + | + |
| 40 | DNB | 41 | Inflammatory/Lactobacillus/Negative for malignancy | − | + | + |
| 41 | SFO | 31 | Unsatisfactory sample for cytology | − | − | − |


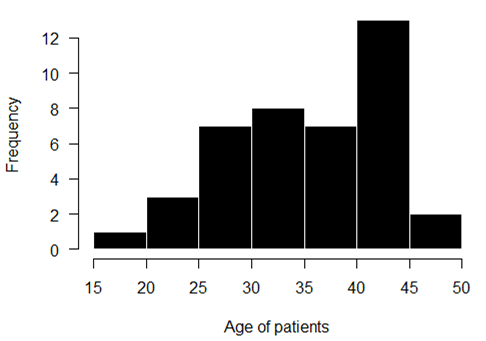


Supplementary Material 3, Graph 1 – Histogram showing the age frequency of the 41 evaluated endocervical samples. The mean age was 35.2 ± 7.8 years, with a range of 19-46 years.
